# Gut microbiota alterations induced by Roux-en-Y gastric bypass result in glucose-lowering by enhancing intestinal glucose excretion

**DOI:** 10.1080/19490976.2025.2473519

**Published:** 2025-03-03

**Authors:** Zhigang Ke, Zongshi Lu, Fan Li, Qingyuan Zhao, Xianhong Jiang, Zhihao Hu, Fang Sun, Zongcheng He, Yi Tang, Qing Li, Stefan van Oostendorp, Xiao Chen, Qiuyue He, Yong Wang, Zhiming Zhu, Weidong Tong

**Affiliations:** a Department of General Surgery, Daping Hospital, Army Medical University, Chongqing, China; b Department of Hypertension and Endocrinology, Daping Hospital, Center for Hypertension and Metabolic Diseases, Chongqing Institute of Hypertension, Army Medical University, Chongqing, China; c Department of Laboratory Animal Science, College of Basic Medical Sciences, Army Medical University, Chongqing, China; d Department of Digestive Medicine, Daping Hospital, Army Medical University, Chongqing, China; e Department of Nuclear Medicine, Daping Hospital, Army Medical University, Chongqing, China; f Department of Surgery, Amsterdam UMC, Vrije Universiteit Amsterdam, Cancer Center Amsterdam, Amsterdam, The Netherlands

**Keywords:** Roux-en-Y gastric bypass, intestinal glucose excretion, gut microbiota, type 2 diabetes mellitus, Biliopancreatic limb

## Abstract

Roux-en-Y gastric bypass (RYGB) results in glucose-lowering in patients with type 2 diabetes mellitus (T2DM) and may be associated with increased intestinal glucose excretion. However, the contribution of intestinal glucose excretion to glycemic control after RYGB and its underlying mechanisms are not fully elucidated. Here, we confirmed that intestinal glucose excretion significantly increased in obese rats after RYGB, which was negatively correlated with postoperative blood glucose levels. Moreover, we also found that the contribution of Biliopancreatic limb length, an important factor affecting glycemic control after RYGB, to the improvement of glucose metabolism after RYGB attributed to the enhancement of intestinal glucose excretion. Subsequently, we further determined through multiple animal models that intestinal glucose excretion is physiological rather than pathological and plays a crucial role in maintaining glucose homeostasis in the body. Finally, we employed germ-free mice colonized with fecal samples from patients and rats to demonstrate that enhanced intestinal glucose excretion after RYGB is directly modulated by the surgery-induced changes in the gut microbiota. These results indicated that the gut microbiota plays a direct causal role in the hypoglycemic effect of RYGB by promoting intestinal glucose excretion, which may provide new insights for developing gut microbiota-based therapies for T2DM.

## Introduction

Type 2 diabetes mellitus (T2DM) is a chronic disease characterized by hyperglycemia that poses a major public health challenge. Although promising therapeutics are being developed, metabolic surgery remains the most effective strategy for treating T2DM at present.^1^ Roux-en-Y gastric bypass (RYGB), one of the classic procedures in metabolic surgery, has been shown to achieve long-term, durable diabetes remission.^2,3^ However, its underlying mechanisms for glucose-lowering have not been fully elucidated. It involves weight-dependent and weight-independent mechanisms. Currently, increasing evidence indicates that weight-independent mechanisms play an important role in this process, such as enhancing insulin secretion, improving β-cell function, promoting intestinal hormone secretion, altering bile acid metabolism and gut microbiota, and enhancing interactions between the gut and other organs (e.g., liver, pancreas, brain).^4–6^ Reprogramming of intestinal glucose metabolism due to intestinal remodeling after RYGB plays an important role in glycemic control improvement, which is manifested by increased intestinal glucose uptake and utilization.^7–9^ Kwon et al.^10^ subsequently found that glucose uptake by the intestine can be excreted into the intestinal lumen after RYGB. This novel phenomenon of intestinal glucose excretion also occurs in diabetic patients^11,12^ and rodents^13^ treated with metformin. Based on these findings, we propose the hypothesis that intestinal glucose excretion may be a potential mechanism for maintaining glucose homeostasis and a key factor in achieving glycemic control after RYGB.^14^

Gastrointestinal rearrangement after RYGB creates three intestinal segments of different lengths, including the alimentary limb (AL), the biliopancreatic limb (BPL), and the common limb (CL). Currently, there is still no consensus on the optimal length of the AL, BPL, and CL that should be bypassed during RYGB due to the significant variation in the total small bowel length (TSBL) between individuals (4–12 m).^15^ Data from human^16–18^ and rodent^19,20^ studies showed that BPL length may be an important factor affecting glycemic control after RYGB. However, there is a paucity of mechanistic studies in this area, with most studies focusing on reporting clinical outcomes. As an influencing factor of glycemic control after RYGB, whether BPL length affects the hypoglycemic effect by modulating intestinal glucose excretion has not been reported.

In recent years, accumulating evidence has suggested that the gut microbiota is considered a potential contributor to either metabolic perturbation or benefits,^21–24^ such as obesity and T2DM. The rearrangement of the gastrointestinal tract after RYGB alters intestinal pH, gastric emptying, intestinal transit time, and bile acid levels, which contribute to significant alterations in the composition of the gut microbiota.^25^ In addition, studies on fecal microbiota transplantation (FMT) from humans^26–28^ and rodents^29^ after RYGB were transplanted into germ-free (GF) animals have further demonstrated that the gut microbiota plays a direct role in the improvement of glucose metabolism after RYGB. Well, it is unclear whether the gut microbiota is associated with increased glucose excretion after RYGB.

To address the questions above, we first confirmed through clinical cohort studies that RYGB can reduce blood glucose levels in patients with T2DM in the long term, independent of weight loss. Subsequently, animal experiments further demonstrated that intestinal glucose excretion is not only a potential for lowering blood glucose after RYGB but also critical for maintaining glucose homeostasis. Finally, we employed GF mice colonized with fecal samples from patients and rats to elucidate the direct contribution of gut microbiota in the enhancement of intestinal glucose excretion after RYGB.

## Results

### RYGB reduces blood glucose in patients with T2DM independent of the degree of weight loss

A total of 92 patients (56 males and 36 females) with T2DM who underwent RYGB were analyzed in this study (Supplementary Table S1). The changes in anthropometric and glycemic parameters after surgery are summarized in Table 1. After surgery, the body weight and fasting plasma glucose (FPG) levels decreased significantly, reaching a minimum at 12 months and remaining essentially stable over the subsequent 48 months. Similarly, glycosylated hemoglobin (HbA1c) also reached its lowest point at 6 months after surgery (8.35 ± 2.02% to 6.12 ± 0.79%, *P* < 0.001). The remission of T2DM was achieved in 67.6%, 57.5%, 48.3%, and 45.1% at 12, 24, 36, and 48 months after surgery. Compared to baseline, the glucose tolerance of T2DM patients improved significantly at 6 months, 12 months, and 36 months after surgery (*P*<0.001, respectively) (Supplementary Figure S1).

**Table 1.** Weight loss outcomes and glycemic control through 48 months after RYGB.

	Baseline	1 month	3 months	6 months	12 months	24 months	36 months	48 months
Follow-up, No. (%)	92	63 (68.4)	56 (60.8)	52 (56.5)	68 (73.9)	40 (43.4)	31 (33.6)	31 (33.6)
Weight (kg)	81.36 ± 20.46	69.92 ± 17.76*	65.88 ± 15.93*	62.96 ± 16.29*	62.06 ± 12.42*	61.22 ± 11.52*	60.19 ± 11.58*	65.16 ± 14.82*
BMI (kg/m^2^	30.02 ± 5.50	26.19 ± 4.76*	24.88 ± 4.43*	23.69 ± 4.18*	23.61 ± 3.53*	23.29 ± 3.10*	23.44 ± 3.21*	24.22 ± 3.96*
FPG (mmol/L)	8.84 ± 3.36	6.25 ± 1.72*	6.00 ± 1.70*	5.71 ± 1.22*	6.15 ± 2.03*	6.03 ± 1.85*	6.33 ± 1.34*	6.11 ± 1.58*
2hPG (mmol/L)	16.67 ± 5.03	7.04 ± 2.87*	6.53 ± 2.74*	5.70 ± 1.93*	6.66 ± 3.07*	6.76 ± 3.31*	7.85 ± 3.48*	8.16 ± 4.16*
HbA1c	8.35 ± 2.02	6.74 ± 0.97*	6.21 ± 0.74*	6.12 ± 0.79*	6.3 ± 1.06*	6.43 ± 0.79*	6.71 ± 1.08*	6.7 ± 0.97*
C-peptide (ng/mL)	1.43 ± 1.44	1.15 ± 1.16	1.17 ± 2.34	0.66 ± 0.37*	0.82 ± 0.44*	1.22 ± 3.02	0.65 ± 0.48*	0.72 ± 0.42*
HOMA-IR	5.00 ± 6.96	2.52 ± 3.12*	1.97 ± 2.51*	1.80 ± 2.36*	2.1 ± 2.7*	1.36 ± 2.23*	1.1 ± 2.51*	1.39 ± 2.83*
Remission, No. (%)				37 (71.1)	46 (67.6)	23 (57.5)	15 (48.3%)	14 (45.1)

BMI, body mass index; FPG, fasting plasma glucose; 2hPG, 2-hour postprandial blood glucose; HbA1c, glycated hemoglobin; HOMA-IR, homeostatic model assessment for insulin resistance. Postoperative follow-up and diabetes remission rates were expressed as No. (%). Data are presented as the mean ± SD. *P < 0.05, compared with baseline.

**Figure 1.**
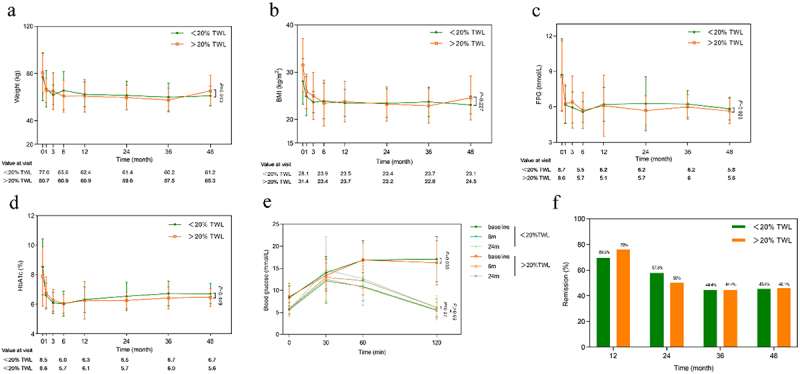
Diabetes remission, OGTT, changes in weight, and glycemic control over 4 years after RYGB between <20%TWL and >20%TWL groups. Body weight (a), BMI (b), FPG (c), and HbA1c (d) were compared between the two groups after surgery. (e) Comparison of preoperative and postoperative OGTT at 6 months and 24 months between the two groups. (f) Comparison of diabetes remission rates in the two groups at each follow-up point. Data are presented as the mean ± SD. *p* values for the overall comparisons were calculated with ANOVA. *Statistically significant *p* value.

BMI, body mass index; FPG, fasting plasma glucose; 2hPG, 2-hour postprandial blood glucose; HbA1c, glycated hemoglobin.

To determine whether the improvement in glucose metabolism after RYGB is associated with postoperative weight loss, the patients were divided into two groups based on a total weight loss (TWL) cutoff point of 20% at 1 year after surgery.^30^ Thirty-two patients achieved more than 20% TWL, while 36 patients experienced less than 20% TWL at the 1-year post-surgery (Supplementary Table S2). Both groups had similar baseline body weight and blood glucose levels. At postoperative follow-up, there were no significant differences in weight changes and glycemic improvement between the two groups after surgery (Figure 1(a–d)). Additionally, there was no notable difference in glucose tolerance between the two groups at 6 and 24 months after surgery (Figure 1(e)). The rate of diabetes remission did not differ between the two groups at 12, 24, 36, and 48 months after surgery (Figure 1(f)). These findings suggest that RYGB can significantly reduce blood glucose in patients with T2DM independent of the degree of weight loss.

### RYGB reduces blood glucose through enhancing intestinal glucose excretion

**Figure 2.**
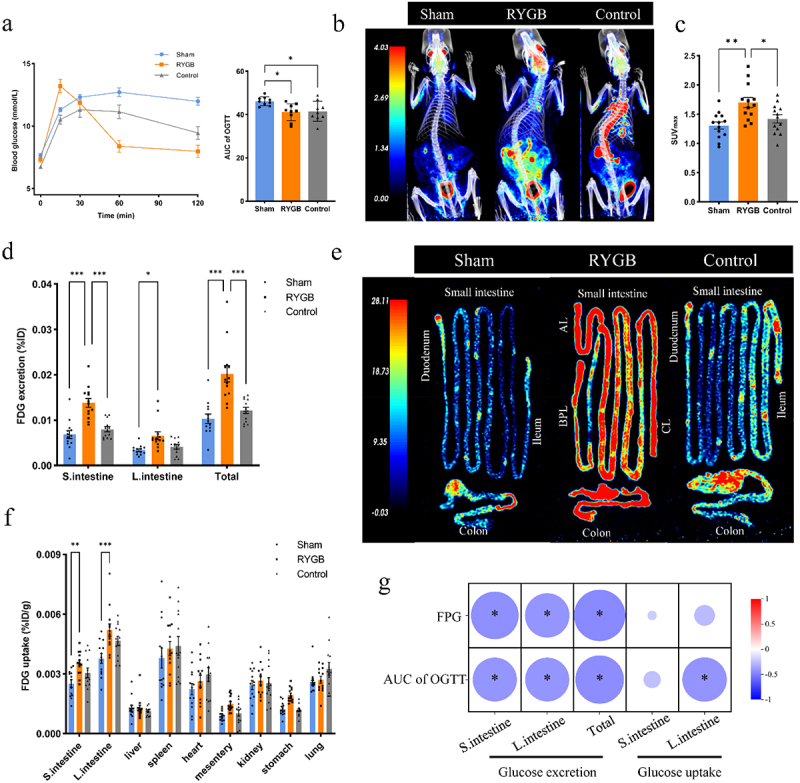
RYGB improves glucose metabolism by enhancing intestinal glucose excretion. (a) Oral glucose tolerance test (OGTT) and area under the curve (AUC) (*n* = 9 rat per group, one-way ANOVA test). (b) Representative whole-body micro- /CT scan images of the sham, RYGB, and control groups at 5 weeks postoperatively, red and blue represent relatively higher and lower FDG accumulation, respectively. (c) The maximum standardized uptake values (SUV_max_) of intestinal FDG uptake in the sham, RYGB, and control groups (*n* = 13 rat per group, one-way ANOVA test). (d) Intraluminal FDG excretion (%ID) in the small intestine and colon in the sham, RYGB, and control groups (*n* = 13 rat per group, two-way ANOVA test). (e) Representative FDG uptake images of the small intestine wall and colon wall of rats in the sham, RYGB, and control groups after PBS lavage, red and blue represent relatively higher and lower values, respectively. (f) FDG uptake (%id/g) in major tissues of rats in the sham, RYGB, and control groups (*n* = 13 rat per group, two-way ANOVA test). (g) Heatmap of the correlations between the FDG uptake and FDG excretion in the small intestine and colon with blood glucose levels (Spearman’s correlation). Data are presented as the mean ± SEM. *P < 0.05, **P < 0.01, ***P < 0.001.

Recent studies found intestinal glucose excretion may be a potential mechanism for improved glucose metabolism independent of weight loss after RYGB.^10,31^ To further investigate the role of intestinal glucose excretion in improving glucose metabolism after RYGB, we performed RYGB or sham surgery on insulin-resistant, diet-induced obese (DIO) Sprague Dawley (SD) rats, and lean rats on a normal diet was given as a comparison (Supplementary Figure S2a). The results showed that impaired glucose tolerance in DIO rats was significantly improved after RYGB and RYGB rats exhibited a greater TWL % (Figure 2(a) and Supplementary Figure S2b). Subsequently, we performed whole-body 18F-fluoro-2-deoxyglucose (FDG) PET/CT scans on DIO rats at 1 and 5 weeks after RYGB. We evaluated FDG accumulation in the intestine (intestinal wall and contents) on SUVmax, a semi-quantitative measure of region-specific accumulation.^11^ SUVmax for the intestine was significantly greater in the RYGB group than in the sham and control groups at 5 weeks postoperatively (Figure 2(b-c)). We also found that SUVmax of the intestine in the RYGB group was higher at 5 weeks postoperatively than at 1 week (Supplementary Figure S2c-d), indicating that the intestinal glucose uptake after RYGB is time-dependent.

To quantify the amount of FDG excreted into the small intestine and colon, we ligated the terminal ileum before administering FDG into the tail vein of DIO rats at 5 weeks postoperatively (Supplementary Figure S2e), whereas the animals were later dissected immediately after PET/CT scanning, and the small intestine and colon were flushed with saline to quantify excreted FDG, respectively. Compared with the sham and control groups, the excretion of FDG in the small intestine of rats was significantly increased after RYGB (0.013 ± 0.0009 vs 0.006 ± 0.0008 vs 0.008 ± 0.0005% injected dose (%ID), *P* < 0.001) (Figure 2(d)). Meanwhile, rats excreted slightly more FDG into the lumen of the colon after RYGB than in the sham-operated group (0.006 ± 0.0008%ID vs 0.003 ± 0.0002%ID, *p* = 0.048) (Figure 2(d)). Multiple organs were also removed and FDG uptake was assessed. The results showed that FDG uptake by the small intestinal wall and colon wall was significantly stronger in the RYGB group than in the sham group (Figure 2(e-f)). Furthermore, we found a negative correlation between intestinal glucose excretion with blood glucose levels (Figure 2(g)). These results indicated that intestinal glucose excretion plays an important role in glycemic control after RYGB.

We then reviewed the PET/CT data of one patient who had undergone RYGB 6 years ago and one patient with newly diagnosed T2DM. Both patients received oral mannitol immediately after intravenous administration of FDG. The results showed strong hypermetabolic activity in the sigmoid colon of the operated patients, characterized by an intense FDG uptake on attenuation-corrected PET images, while no abnormal uptake was found in the control subject (Supplementary Figure S2f).

### Enhanced intestinal glucose excretion is more pronounced in RYGB with a long-BPL

Previous studies have suggested that BPL length may be an important factor affecting glycemic control after RYGB.^19,32^ To elucidate in depth the contribution of intestinal glucose excretion to glycemic control after RYGB, we hypothesized that RYGB with long BPL may exert better glycemic control by promoting more intestinal glucose excretion. To demonstrate this hypothesis, we constructed an RYGB model with different ratios of the BPL and AL and a constant CL on DIO rats (Supplementary Figure S3a). The median BPL length in the long-BPL (L-BPL) group and the short-BPL (S-BPL) group were 39.63 ± 5.34 cm and 11.56 ± 4.69 cm, and the proportions of BPL to TSBL were 34.8% and 11.1%, respectively (Supplementary Figure S3b-c). Compared with the sham group and S-BPL group, DIO rats in the L-BPL group showed a significant reduction in body weight after surgery (Supplementary Figure S3d-e). At 3 and 6 weeks after surgery, the glucose tolerance of rats in the L-BPL group was significantly improved compared with the S-BPL group, while there was no significant different in insulin tolerance between the two groups (Supplementary Figure S3f-i).

Subsequently, we divided the patients in the above cohort study into two groups based on the length of BPL to assess whether BPL length affects glycemic control in patients with T2DM after RYGB. The S-BPL group included 45 patients who received a 50-cm length of BPL, while the L-BPL group consisted of 47 patients who received 100-cm limbs (Supplementary Table S3). After surgery, patients in the L-BPL group experienced more weight loss and greater changes in BMI than those in the S-BPL group (Supplementary Figure S4a-b). FPG levels showed more significant improvement in the L-BPL group compared to the S-BPL group (Supplementary Figure S4c), while there was no significant difference in the change of HbA1c between the two groups (Supplementary Figure S4d). These results suggest that RYGB with a longer BPL can result in better glycemic control.

**Figure 3.**
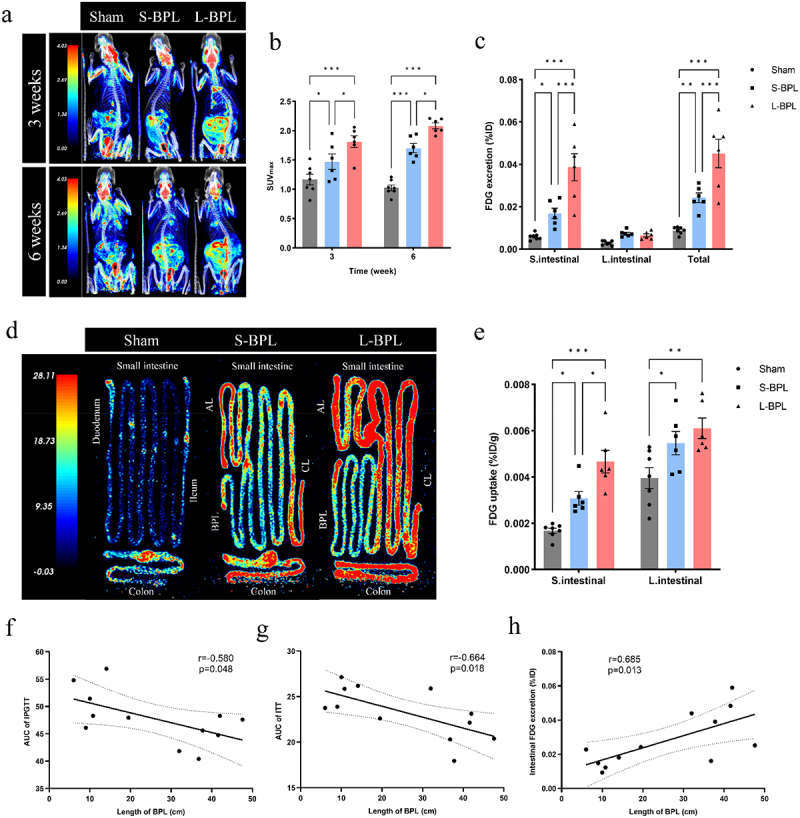
Enhanced intestinal glucose excretion is more pronounced in RYGB with a long BPL. (a) Representative whole-body images obtained from micro-pet/ct scans of rats in the sham, S-BPL and L-bpl groups at 3 and 6 weeks postoperatively, red and blue represent relatively higher and lower FDG accumulation, respectively. (b) SUV_max_ of intestinal FDG uptake in the sham, S-BPL and L-bpl groups (*n* = 6–7 rat per group). (c) Intraluminal FDG excretion (%ID) in small intestine and colon in the sham, S-BPL and L-bpl groups (*n* = 6–7 rat per group). (d) Representative FDG uptake image of small intestine wall and colon wall of rats in the sham, S-BPL and L-bpl groups after PBS lavage at 6 weeks postoperatively, red and blue represent relatively higher and lower values, respectively. (e) FDG uptake (%id/g) in the small intestine wall and colon wall of rats in the sham, S-BPL and L-bpl groups (*n* = 6–7 rat per group). (f to h) correlation between BPL length with intraperitoneal glucose tolerance testing (IPGTT) (f), insulin tolerance test (ITT) (g), and intestinal FDG excretion (h) (Spearman’s correlation). Data are presented as the mean ± SEM. The P-values for b, c, and e were calculated by two-way ANOVA test.*P < 0.05, **P < 0.01, ***P < 0.001.

To evaluate the impact of the BPL length on intestinal glucose excretion after RYGB, we performed a whole-body PET-CT examination on rats in both groups at 3 and 6 weeks postoperatively. The results showed that SUVmax of the intestine in the L-BPL group was significantly higher than that in the S-BPL group and the sham group at 3 and 6 weeks postoperatively (Figure 3(a-b)). At 6 weeks postoperatively, we dissected the intestines and washed the small intestine and colon with saline respectively to quantify the excreted FDG, and found that the excreted FDG in the small intestine of the rats in the L-BPL group was significantly more than that in the S-BPL group, whereas the FDG excreted in the colon did not different significantly between the two groups (Figure 3(c)). We also observed that the FDG uptake in the small intestine wall of rats in the L-BPL group was stronger than that in the S-BPL group, whereas FDG uptake in the colon wall was not significantly different (Figure 3(d-e)). Pearson correlation analysis showed that BPL length was negatively correlated with the area under curve (AUC) of intraperitoneal glucose tolerance testing (IPGTT) and AUC of insulin tolerance testing (ITT) and positively correlated with intestinal glucose excretion (Figure 3(f-h)). These results further confirmed that intestinal glucose excretion is crucial in glycemic control after RYGB.

### Intestinal glucose excretion is the key to maintaining glucose homeostasis

**Figure 4.**
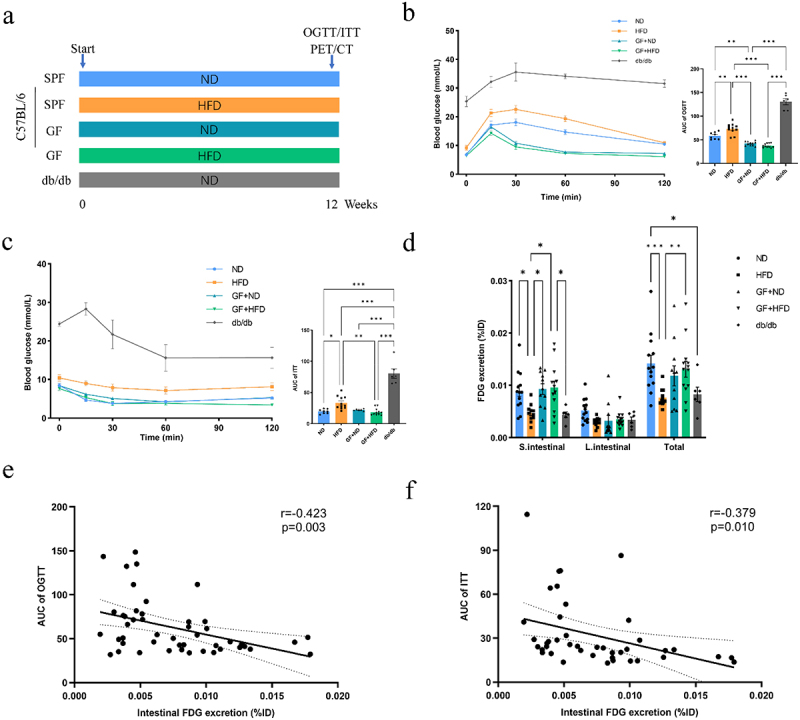
An increase in intestinal glucose excretion is associated with better oral glucose tolerance. (a) Schematic diagram of animal experiment design. (b) Oral glucose tolerance test (OGTT) and area under the curve (AUC) (*n* = 6–11 mice per group, one-way ANOVA test). (c) Insulin tolerance test (ITT) and AUC (*n* = 6–11 mice per group, one-way ANOVA test). (d) Intraluminal FDG excretion (%ID) in the small intestine and colon of mice in the ND, HFD, GF+ND, GF+HFD, and db/db groups (*n* = 6–13 mice per group, two-way ANOVA test). (e, f) Correlation between intestinal FDG excretion with AUC of OGTT (e) and AUC of ITT (f) (Spearman’s correlation). Data are shown as means ± SEM. *P < 0.05, **P < 0.01, ***P < 0.001.

To further elucidate whether intestinal glucose excretion is an important contributor to maintaining glucose homeostasis, we performed whole-body PET-CT scans to clarify the relationship between intestinal glucose excretion and blood glucose levels on specific pathogen-free (SPF) mice receiving normal diet (ND) or high-fat diet (HFD), germ-free (GF) mice receiving ND or HFD, and db/db mice receiving ND (Figure 4(a)). The results showed that the db/db mice were significantly hyperglycemic, and their glucose tolerance and insulin tolerance were significantly impaired (Figure 4(b-c)). SPF mice fed an HFD showed significantly impaired glucose tolerance and insulin tolerance compared with SPF mice fed a normal diet, whereas GF mice showed resistance to impaired glucose tolerance and insulin tolerance induced by HFD (Figure 4(b-c)). PET/CT showed that HFD-fed SPF mice and db/db mice had significantly lower intestinal FDG excretion compared to SPF mice fed a normal diet (Figure 4(d)). In contrast, intestinal FDG excretion was significantly greater in GF mice fed an HFD than in SPF mice fed an HFD (Figure 4d). Interestingly, there were no significant differences in glucose tolerance and intestinal FDG excretion in GF mice fed ND or HFD (Figure 4(b-d)). We also found no significant difference in small intestinal wall FDG uptake between SPF mice, GF mice, and db/db mice, whereas the colon wall FDG uptake was higher in GF mice than in SPF mice and db/db mice (Supplementary Figure S5a-b). Pearson’s correlation analysis indicates that intestinal glucose excretion was negative with the AUC of oral glucose tolerance testing (OGTT) (Figure 4(g)) and the AUC of ITT (Figure 4h). These results further indicate that intestinal glucose excretion may be an important mechanism for maintaining glucose homeostasis, and it is closely related to the gut microbiota.

### RYGB results in significant alterations in the composition of the gut microbiota

**Figure 5.**
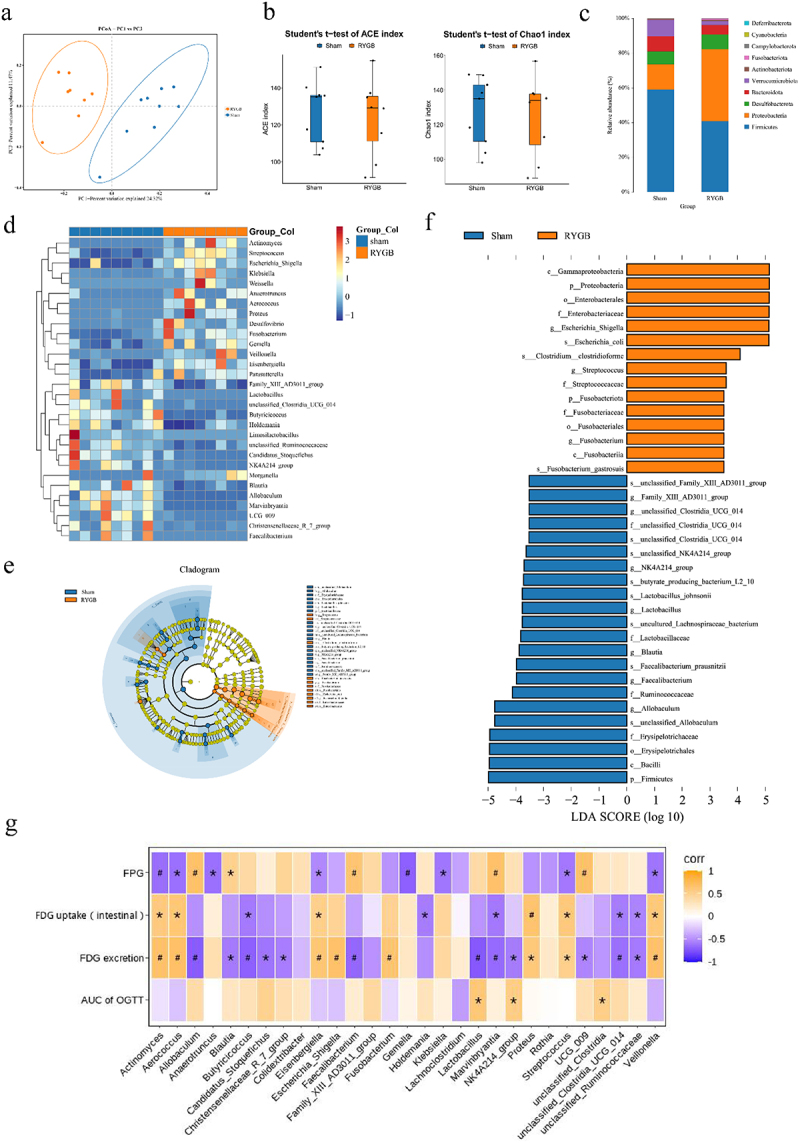
RYGB alters the composition of gut microbiota. (a) Principal coordinate analysis (PCoA) of all samples by Binary-Jaccard distance in the sham and RYGB groups. (b) Alpha-diversity of the gut microbiota between the sham and RYGB groups, as indicated by the ACE and Chao1 indices. (c) Mean relative abundance of bacterial phylum in the sham and RYGB groups. (d) Heatmap of different bacteria at the genus level in the sham and RYGB groups based on the 16S rDNA gene sequencing analysis. (e) Taxonomic cladograms generated from LEfSe analysis of 16S rRNA sequences. Each circle’s size is proportional to the taxon’s abundance. (f) LDA score representing the taxonomic data with significant difference between the sham and RYGB groups. Only LDA > 4 are shown. (g) Heat map of the correlation between the relative abundance of bacterial genus and glucose excretion and glucose levels. *P < 0.05, #*P* < 0.01.

The above data indicated that intestinal glucose excretion is correlated with gut microbiota. Previous studies have indicated that RYGB can remodel the gut microbiota.^33,34^ To this, we hypothesized that gut microbiota might mediate the enhancement in intestinal glucose excretion after RYGB. Thus, we applied 16S rDNA based on PacBio three-generation full-length sequencing of fecal samples from DIO rats 5 weeks after RYGB. Principal coordinate analysis (PCoA) of UniFrac distances (beta-diversity) showed a clear separation of gut microbiota clusters between the sham and RYGB groups (Figure 5(a)). However, there was no difference between the two groups in terms of alpha diversity (Figure 5(b)). There are significant differences at the phylum and genus levels of gut microbiota between the sham and RYGB groups. At the phylum level, the RYGB group had higher Proteobacteria and Fusobacteriota and lower Firmicutes and Verrucomicrobiota than in the sham group (Figure 5(c)). At the genus level, the relative abundances of Holdemania, Allobaculum, and Faecalibacterium were decreased, while the abundance of Streptococcus, Escherichia-Shigella, Aerococcus, and Fusobacterium were enhanced in RYGB group (Figure 5(d)). Based on linear discriminant analysis (LDA) effect size (LEfSe) analysis, we found that the sham group was characterized by enriched Faecalibacterium, Blautia, and Allobaculum, while Escherichia-Shigella, Streptococcus Fusobacterium were dominant bacterial in the RYGB group (Figure 5(e-f)). Correlation analysis showed that Aerococcus, Eisenbergiella, Streptococcus, and Veillonella were positively correlated with intestinal glucose uptake and intestinal glucose excretion but negatively correlated with FPG (Figure 5(g)).

### Fecal microbiota transplantation in germ-free mice recapitulates the enhanced intestinal glucose excretion after RYGB

**Figure 6.**
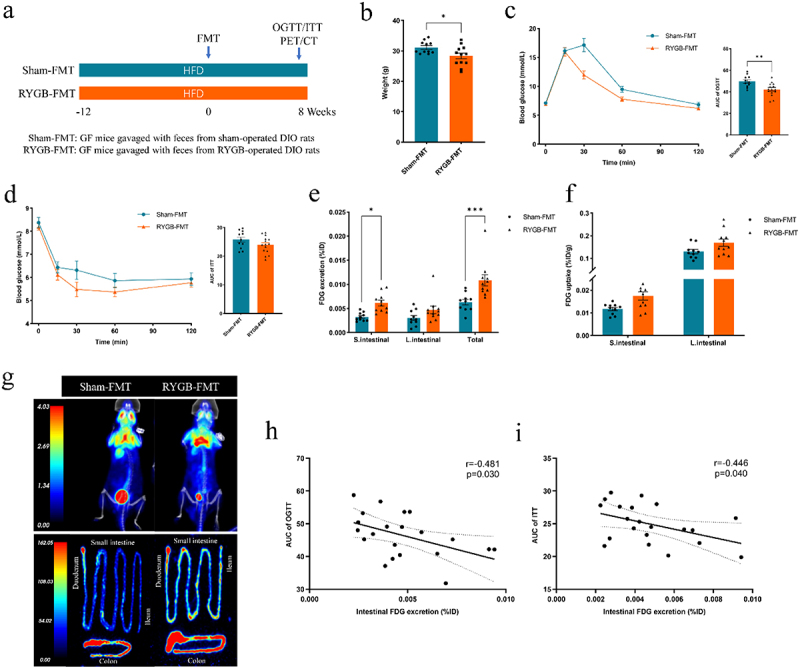
The transplantation of the gut microbiota from RYGB-treated rats to GF mice can enhance intestinal glucose excretion and improve glucose metabolism. (a) Schematic diagram of animal experiment design. (b) Body weight of recipient mice in the sham and RYGB groups at 8 weeks after fecal microbiota transplantation (*n* = 11–12 per group, unpaired Student t-test). (c) Oral glucose tolerance test (OGTT) and area under the curve (AUC) (*n* = 11–12 per group, one-way ANOVA). (d) Insulin tolerance test (ITT) and AUC (*n* = 11–12 per group, one-way ANOVA). (e) Intraluminal FDG excretion (%ID) in the small intestine and colon of mice in the sham-FMT and RYGB-FMT groups (*n* = 10–11 per group, two-way ANOVA). (f) FDG uptake in the small intestine wall and colon wall of mice in the sham-FMT and RYGB-FMT groups after PBS lavage (*n* = 10–11 per group, two-way ANOVA). (g) Representative whole-body and FDG uptakes image of the small intestine wall and colon wall of mice after PBS lavage in the sham-FMT and RYGB-FMT groups. (h, i) correlation between intestinal FDG excretion with AUC of OGTT (h) and AUC of ITT (i) (Spearman’s correlation). Data are shown as means ± SEM. *P < 0.05, **P < 0.01, ***P < 0.001.

To further investigate the causal relationship RYGB-induced gut microbiota alteration and the intestinal glucose excretion, we performed FMT in GF mice. GF mice were gavaged with cecum contents from DIO rats after sham surgery or RYGB (Figure 6(a)). Compared with the sham-FMT group, recipient mice in the RYGB-FMT group had lower body weight (Figure 6(b)) and better glucose tolerance and insulin tolerance (Figure 6(c-d)). It is important to note that the intestinal FDG excretion of recipient mice in the RYGB-FMT was significantly higher than that of the sham-FMT group (Figure 6(e)), although there was no significant difference FDG uptake in the small intestine wall and colon wall of mice between the two groups (Figure 6(f-g)). Pearson’s correlation analysis indicates that intestinal glucose excretion in recipient mice after FMT was negatively with the AUC of OGTT and AUC of ITT (Figure 6(h-i)).

**Figure 7.**
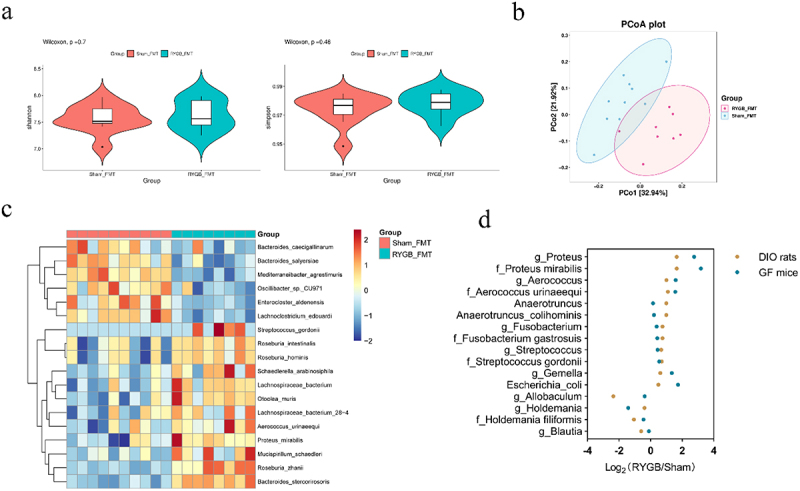
Alteration of gut microbiota in germ-free mice with fecal microbiota transplantation from RYGB. (a) Alpha-diversity of the gut microbiota between the sham-FMT and RYGB-FMT groups, as indicated by the Shannon and Simpson indices. (b) PCoA of all samples by Bray-Curtis distance in the sham-FMT and RYGB-FMT groups. (c) Heatmap of different bacteria at the species level in the sham-FMT and RYGB-FMT groups based on the metagenomic sequencing analysis. (d) Consistent alteration in bacteria abundance in the donor rats and recipient mice models. The fold change abundance between sham and RYGB was calculated.

Next, we performed metagenomic sequencing of the cecum contents of recipient GF mice to explore the colonization of RYGB-altered microbiota. Similar to the donor rats, there was no significant difference in alpha diversity between the sham-FMT and RYGB-FMT groups (Figure 7(a)). Beta diversity analysis of PCoA using Bray-Curtis distance again showed significant segregation of gut microbiota between the two groups (Figure 7(b)). Further analysis revealed 18 significantly different abundant bacteria at the species level (Figure 7(c)). We further analyze the consistence of microbiota alterations between the donor rats and recipient mice models. We found that bacteria with increased abundance in RYGB rats compared with sham rats were consistently increased in RYGB-FMT mice compared with sham-FMT mice, whereas bacteria with decreased abundance in RYGB rats were consistently reduced in RYGB-FMT mice (Figure 7(d)). These data show that RYGB resulted in significant alterations in the composition of the gut microbiota, and this alteration is partially reflected in GF mice colonized with RYGB microbiota.

We also established a human microbiota-associated (HMA) mouse model by transplanting fecal microbiota from six donors, including three patients after RYGB and three obesity patients without RYGB (Supplementary Figure S6a). Clinical and biochemical measurements of these two groups of patients are summarized in Supplementary Table S4. After microbiota transplantation, there was no difference in body weight between the two groups (Supplementary Figure S6b). However, GF mice colonized with microbiota from patients undergoing RYGB had better glucose tolerance and insulin tolerance (Supplementary Figure S6c-d). In addition, we observed significantly higher intestinal FDG excretion in GF mice colonized with microbiota from patients undergoing RYGB than in mice colonized with microbiota from obese patients (Supplementary Figure S6e). However, there was no significant difference in FDG uptake in the small intestine wall and colon wall of mice between the two groups (Supplementary Figure S6f-g). These results suggest that the gut microbiota is essential for intestinal glucose excretion after RYGB.

## Discussion

In this study, we report three important findings based on clinical studies and animal experiments. First, we note that intestinal glucose excretion is not only critical for lowering blood glucose after RYGB but also a potential mechanism for maintaining glucose homeostasis in the body. Second, RYGB with a long BPL limb achieves better glycemic control, which was partly attributed to the enhancement of intestinal glucose excretion. Finally, we found that enhanced intestinal glucose excretion after RYGB is directly modulated by the surgery-induced changes in the gut microbiota.

Previously, it was generally accepted that the ameliorative effect of RYGB on glucose metabolism was closely related to weight loss.^35^ However, as the body of preclinical and clinical evidence has grown, it has become apparent that weight loss explanations may play a role, it does not fully account for the improvement in glucose homeostasis after surgery.^36^ Our previous study also showed that RYGB improved glucose metabolism in T2DM patients with low BMI.^37–39^ In a subgroup analysis based on a 20% TWL threshold, we found no significant difference in glycemic control and improvement in diabetes remission between the two groups. In recent years, the gut has been widely recognized as an important organ for RYGB to exert weight-independent hypoglycemic mechanisms involving gut hormones, bile acids, intestinal gluconeogenesis, gut microbiota, and signaling interaction between the gut and other organs.^40^ Although most studies on the mechanisms by which RYGB alleviates T2DM have focused on gut hormones,^41^ direct glucose handling by the gut is also an important contributor to glucose homeostasis. Studies involving humans and rodents have demonstrated an increased intestinal glucose uptake and utilization after RYGB,^7,8^ and this increased intestinal glucose uptake is significantly negatively associated with fasting glucose levels in patients undergoing gastrectomy and RYGB.^9,42^ Our findings also showed that the intestinal glucose uptake in the DIO rats at 5 weeks after RYGB was higher than that at 1 week postoperatively, indicating that the enhancement of intestinal glucose uptake after RYGB has a time-dependent manner.

Kwon et al.^10^ observed migration of the site of increased intestinal glucose uptake in repeat PET/CT scans performed on the same day in patients after gastrectomy, prompting a hypothesis that glucose uptake in the intestine might be excreted into the intestinal lumen. To test this hypothesis, they performed PET/CT scans on sham-operated and post-RYGB rats and washed the intestinal lumen to quantify excreted FDG radioactivity. The results revealed a significant enhancement in intestinal glucose excretion in the AL and CL of the RYGB group compared to the sham group. In a separate study, Yasuko et al. found by PET-MRI that patients with T2DM who were treated with metformin had a 3- to 4-fold higher FDG accumulation in the lumen of the ileum and colon than the control group.^11^ These studies suggest that intestinal glucose excretion is significantly increased after either gastric bypass or metformin treatment. However, the precise location of intestinal glucose excretion and its contribution to hypoglycemia deserves further investigation. Our findings further revealed that RYGB increased glucose excretion not only in the small intestine but also in the colon, and the amount of intestinal glucose excreted is negatively correlated with blood glucose levels. To further identify whether intestinal glucose excretion is physiological, we added lean rats on a normal diet as a control group. The results showed that even lean rats that did not undergo RYGB exhibited a certain level of intestinal glucose excretion, which is consistent with previous observations in rodents and humans.^43–45^ This finding suggests that the intestinal glucose excretion after RYGB may be physiological rather than pathological. Meanwhile, we also found that the intestinal glucose excretion in lean rats was slightly more than that in the sham group, although there was no statistically significant difference. This suggests that high-fat-diet-induced obesity may cause a worsening of blood glucose by inhibiting intestinal glucose excretion. To validate this hypothesis, we compared the intestinal glucose excretion between lean mice on a normal diet and obese mice on a high-fat diet and found that the intestinal glucose excretion of obese mice was significantly lower than that of lean mice, and the glucose tolerance of obese mice was significantly impaired. We observed intense FDG uptake in the sigmoid colon of a patient who had undergone RYGB and was instructed to ingest mannitol (an osmotic laxative that can be used for bowel preparation) before PET/CT examination. However, a colonoscopy performed on this patient nearly a month ago revealed no abnormalities in the colon. This suggests that circulating FDG may be transferred through the intestinal tract to the intestinal lumen and subsequently excreted into the sigmoid colon under the influence of colonic peristalsis.

In the pursuit of maximum glycemic control after RYGB, multiple studies have attempted to explore the effect of different limb lengths on glycemic improvement. However, there is no consensus on the optimal length of the bypassed small bowel segments because there are three segments of limbs that can be manipulated (AL, BPL, and CL) and significant differences in total small bowel length between individuals. Nevertheless, multiple cohort studies have suggested that longer BPL limbs may achieve better glycemic control after RYGB,^16,17^ which has been confirmed in a mouse model with fixed small intestine length.^19,20^ Our results show that patients with longer BPL limbs experienced a more pronounced decrease in fasting blood glucose compared to those in the short BPL group, although there was no significant difference in HbA1c and diabetes remission rates between the two groups. Furthermore, our animal experiment also confirmed that the postoperative glucose control of rats in the long BPL group was significantly higher than that in the short BPL group, which is consistent with the results of Schneider’s study.^19^ Notably, the underlying mechanisms of increased glycemic improvement due to longer BPL remain unclear. Previous studies have suggested that undigested nutrients bypassing the longer foregut (BPL limb) and entering directly into the ileum may stimulate more gut enteroendocrine responses and gut hormone secretin, such as glucagon-like peptide-1 (GLP-1).^46,47^ However, Miras et al.^48^ found no effect of BPL limb length on GLP-1 secretion. Another rodent study suggested that a longer BPL could decrease intestinal glucose absorption and increase intestinal utilization as well as GLP-1 secretion.^49^ Given that intestinal glucose excretion is a potential mechanism for glycemic control after RYGB, we hypothesize that RYGB with an extended BPL might achieve better glucose control by inducing more intestinal glucose excretion. The results showed that DIO rats in the long BPL group had significantly more intestinal glucose excretion than those in the short BPL group, which is consistent with the trend of blood glucose improvement in both groups. To the best of our knowledge, this is the first article to examine the effect of the BPL limb length on intestinal glucose excretion. The findings also further confirm the important role of intestinal glucose excretion in glycemic control after RYGB.

There is growing evidence that gastrointestinal reconstruction after RYGB dramatically alters the intestinal microenvironment, leading to significant changes in the composition and diversity of the gut microbiota.^50–54^ FMT experiments in which human and rodent animal feces were transplanted into GF mice have further demonstrated that the gut microbiota plays a direct causal role in the improvement of glucose metabolism after RYGB.^28,29,53^ However, the mechanisms by which the gut microbiota acts as an endogenous regulator of improved glucose metabolism after RYGB remain poorly understood. It has been shown that improvements in glucose homeostasis mediated by alterations in gut microbiota after bariatric surgery may be associated with attenuated intestinal glucose absorption, increased brown fat thermogenesis, and reduced adipose tissue inflammation.^27–29,55^ In this study, we first compared the intestinal glucose excretion of SPF mice and GF mice fed with an HFD. The results showed that the intestinal glucose excretion was significantly higher in GF mice than in SPF mice, and that the GF mice were resistant to impaired glucose tolerance induced by the HFD. Subsequently, GF mice that received colonization with rats or human fecal microbiota after RYGB exhibited significantly increased intestinal glucose excretion, and the characteristics of glucose metabolism in the FMT donors were transferred to the GF recipient mice. Finally, we analyzed changes in the composition of the cecum microbiota in donor rats before FMT and in recipient GF mice after FMT. The results showed that RYGB significantly altered the composition of the gut microbiota and these changes were partially transferred to GF mice colonized with RYGB microbiota. Gut microbiota composition was also found to be profoundly altered in rats with long BPL length compared to rats with short BPL length.^20^ We also observed an increase in the relative abundance of Mucispirillum schaedleri (phylum: Deferribacteres, genus: Mucispirillum) in GF mice that received colonization with the RYGB microbiota, which is consistent with the findings of Münzker et al.^29^ These findings suggest that enhanced intestinal glucose excretion after RYGB may be driven by specific gut microbial characteristics.

This study has several limitations. First, whether the rapid improvement of early blood glucose after RYGB is related to intestinal glucose excretion remains unclear. Second, although our and previous studies have found that RYGB increases FDG accumulation in the intestinal lumen of rodents^10^, there is still a lack of population-based evidence for this new phenomenon, and future studies need to require the application of non-conventional methods to quantify the total absolute amount of radioactivity in the intestine, such as PET-MRI enterography.^56^ Finally, we found that the main site of intestinal glucose excretion is the small intestine, and the small intestinal microbiota has been reported to play a role in the regulation of glucose metabolism.^57^ Therefore, further studies should focus on the role of the small intestinal microbiota in regulating intestinal glucose excretion. In addition, Glucose transmembrane transport in mammalian cells requires the mediation of glucose transporter proteins (GLUTs). The gut microbiota can regulate the expression of GLUTs,^58^ and the absence of GLUTs affects the composition of the gut microbiota.^59^ It has been shown that increased intestinal glucose excretion after RYGB is mainly mediated by the upregulation of basolateral and apical membrane GLUT1 expression in small intestinal enterocytes.^10^ Then, whether and how the gut microbiota promotes intestinal glucose excretion by regulating GLUT1 expression and the specific bacteria species involved remain to be elucidated in depth, which is also our ongoing research program.

In conclusion, our study reveals that intestinal glucose excretion is not only a potential mechanism for lowering blood glucose after RYGB but is also critical for maintaining glucose homeostasis. We also propose for the first time that increased intestinal glucose excretion is directly modulated by surgery-induced changes in the gut microbiota. This study provides a new perspective for understanding the mechanism of RYGB in the treatment of T2DM, providing new ideas for the development of new anti-diabetes therapeutic strategies in the future.

## Materials and methods

### Study cohort

Ninety-two patients with T2DM who underwent RYGB in our institution from May 2010 to June 2020 were retrieved from a prospectively collected database. Patients who failed to follow up were excluded. The follow-up rates were 68.4% at 1 month, 60.8% at 3 months, 56.5% at 6 months, 73.9% at 12 months, 43.4% at 24 months, 33.6% at 36 months, and 33.6% at 48 months. They were divided into two groups according to BPL length: the short BPL (S-BPL) group, which consisted of a BPL of 50 cm and an alimentary limb of 150 cm; and the long BPL (L-BPL) group, which consisted of a BPL of 100 cm and alimentary limb of 100. All patients met the Chinese guidelines (2014) developed by Chinese society for metabolic & bariatric surgery. The study was approved by the Ethics Committee and institutional review at our hospital and was compliant with the principles of the Helsinki Declaration.

### Animals and treatments

Animal experiment 1. To elucidate the contribution of intestinal glucose excretion to glycemic control after RYGB. SD rats weighting 180–200 g were fed a HFD (60%kcal fat; research diets, catalog no. D12492) for 12 weeks to induce obesity. These rats were housed under a 12-hour day-night cycle with free access to food and water. They were then randomly assigned to either RYGB (S-RYGB and L-RYGB) or sham surgery. Animals were fasted overnight before operation. During surgery, Anesthesia was induced and maintained with isoflurane (4% induction and 1.5% maintenance). Animals were fully fasted (no food or water) for the first 24 h after surgery. After assessing the health and behavior of each animal, a limited liquid diet was reintroduced 24–72 h and transition to a solid diet was initiated 72 h after operation.^60^ On postoperative day 7, animals were provided solid HFD. PET/CT scans were performed at 5 weeks postoperatively. Immediately after PET/CT scanning, the animals were executed and we dissected the intestines and washed the small intestine and colon with PBS respectively to quantify the excreted FDG.

Animal experiment 2. To determine the effect of intestinal glucose excretion on glucose homeostasis. Prior to the PET/CT scan, 8-week-old C57BL/6 mice were housed in SPF or GF conditions. They were received ND or HFD for 12 weeks, respectively. db/db mice were received normal diet. Immediately after PET/CT scanning, the animals were executed and we dissected the intestines and washed the small intestine and colon with saline respectively to quantify the excreted FDG.

Animal experiment 3. To further investigate whether the altered gut microbiota after RYGB directly affects intestinal glucose excretion. GF mice were randomized to colonize with fecal samples from patients and rats.

Germ-free mice were bred at the Department of Laboratory Animal Science, The Army Medical University. The animal experiments were performed in compliance and were approved by the Ethics Committee of The Army Medical University.

### Surgical technique

For the cohort study, laparoscopic RYGB was performed by the same surgical team and used a 5-port technique. The gastric pouch was approximately 30 ml. In the L-BPL group, the jejunum was divided at 100 cm from the Treitz’s ligament (BPL), and the distal end was gastro-jejunal anastomosed to the gastric pouch, and then the jejuno-jejunal anastomosis is performed at 100 cm from the gastro-jejunal anastomosis (AL). Whereas the length of the BPL was 50 cm and the AL was 150 cm in the L-BPL group. The mesenteric and Petersen’s defects were closed.

For the animal study, standard RYGB was performed using an open approach with a dissection incision to expose the abdominal cavity. The Treitz’s ligament was identified and the jejunum was transected 15 cm downstream of this ligament. The stomach was transected 3 mm aboral to the gastroesophageal junction, and the distal gastric incision was sutured. The distal jejunum was anastomosed to the stomach with 8–0 silk. An end-to-side anastomosis of the remaining proximal jejunum with the small intestine 20 cm distal to the gastrojejunostomy was performed. The laparotomy was closed with a 4–0 silk suture placed in two layers.^61^ In the study of the effect of different BPL lengths on intestinal glucose excretion, we measured the total length of the small intestine and fixed the CL branch at 60 cm. in the L-BPL group, the BPL length was set to 70% of the remaining small intestine, while it was set to 30% in the S-BPL group. Sham operation was performed as a control. The sham operation consisted of laparotomy, jejunal transection, and sutured. The exposure time of the abdominal cavity during the operating was the same as that of the RYGB operation.

### Remission of diabetes

According to an expert consensus jointly organized by the American Diabetes Association (ADA) and European Association for the Study of Diabetes (EASD),^62^ remission was defined as an HbA1C level<6.5% measured at least 3 months after cessation of antidiabetic medication.

### Metabolic assay

An oral glucose tolerance testing (OGTT) was performed in rat after overnight fasting (12 hours). Blood glucose levels were measured from the tail vein at 0, 15, 30, 60, and 120 min with an AutoCoding glucometer (CareSens) after oral gavage of 2 g/kg body weight of glucose.

For the intraperitoneal glucose tolerance testing (IPGTT), glucose (2 g/kg body weight) was administered via an intraperitoneal injection after overnight fasting, the blood glucose levels were measured from the tail vein at 0, 15, 30, 60, and 120 min with an AutoCoding glucometer.

For the insulin tolerance testing (ITT), insulin (0.5 U/kg) was administered via intraperitoneal injection after 4 h fasting. The glucose levels of the tail vein were measured at 0, 15, 30, 60, and 120 min after insulin load.

### PET/CT scan and image analysis

Animals were fasted overnight before 18F-fluoro-2-deoxyglucose (FDG) injection. 18F-FDG was injected intravenously via the tail vein (dose:500uCi in rats and 1mCi in mice). After 40 min of uptake (60 min in mice), animals were anesthetized with 1% isoflurane and static PET scans were performed using a micro-PET/CT of small animals. A CT scan was acquired following PET scans for anatomical localization, attenuation, and scatter corrections. Immediately after the PET/CT scan, the animals were euthanized and their organs were removed and weighed, and the radioactivity was measured using a γ-counter. FDG organ biodistribution data were corrected for attenuation according to the time of anesthesia and normalized for tissue mass (g) and the radioactivity levels upon injection. The results were expressed as %ID/g. To quantify intestinal FDG excretion, we flushed the lumen of the small intestine and colon of rats with 150 ml (100 ml for mice) of saline. Gama counts were performed on the small intestine or colon before and after flushing the lumen. The FDG uptake by the intestinal wall after intestinal lumen flushing is the FDG uptake by the intestinal epithelial tissue, whereas the intestinal FDG excretion is the FDG value of the intestinal contents after flushing. Finally, the flushed small intestine and colon were scanned again to obtain PET images. When measurements of intestinal excretion of FDG, the counts per minute were normalized to the injected dose of radioactivity and expressed in units of %ID.

### Fecal microbiota transplantation

The rats were necropsied at 4 weeks after sham and RYGB, and the contents of the cecum were collected and stored at −80°C with 20% sterile glycerol. During FMT, the cecum contents were suspended in sterile saline for homogenization (40 mg/ml) and then centrifuged (600 g, 5 min) to obtain the supernatant. The supernatant was immediately introduced into the isolator, and each GF mice were received a gavage with 200ul supernatant for 5 consecutive days. For human fecal microbiota transfer, fecal samples were obtained from three patients 7 years after RYGB and from patients suffering from obesity with impaired glucose tolerance. The steps for preparation and transplantation of fecal samples were performed as described in the FMT section above.

### 16S rDNA gene sequencing

Bacterial genomic DNA was extracted from cecum contents samples by TGuide S96 Magnetic Stool DNA Kit (Tiangen Biotech (Beijing) Co., Ltd, China). DNA quality and quantity were assessed by agarose gel electrophoresis and ultraviolet spectrophotometer. The full-length of bacterial 16S rDNA genes were amplified using barcoded conserved primers 27 F (5'- AGRGTTTGATYNTGGCTCAG-3’) and 1492 R (5’-TASGGHTACCTTGTTASGACTT-3’). The amplified library was sequenced using a PacBio SMRT RS II DNA sequencing platform (Pacific Biosciences, Menlo Park, CA, USA).

### Metagenomic sequencing

DNA was extracted from samples using the BioTeke Fecal Genome DNA Extraction Kit (AU46111–96, China). Sequencing libraries were generated by TruSeq Nano DNA Library Preparation Kit-Set for Illumina (#FC-121–4001, USA), adhering to the manufacturer’s recommendations. The metagenome libraries were sequenced on an Illumina NovaSeq 6000 platform with PE150. For metagenomic sequencing and analysis, sequencing adapters and low-quality reads were discarded from de-multiplexed raw sequences, and high-quality reads that remained were then assembled de novo for each sample using the MEGAHIT (version 1.2.9). The coding regions (CDS) of the assembled contigs were predicted using MetaGeneMark and the CDS sequences of all samples were clustered using CD-HIT to obtain unigenes. Taxonomic assessment of microbiota based on the NR database using Diamond (v 0.9.14).

### Statistical analysis

All graphs were produced and statistical analysis was performed by the GraphPad Prism 9.0 software. Continuous variables were expressed in terms of means ± standard deviation (SD) or standard error of the mean (SEM). Categorical variables were presented as numbers (percentages). Continuous variables in the different groups were tested for normal distribution using the Shapiro-Wilk test. The significant difference between the two groups was calculated using the unpaired T-test or the Mann-Whitney test depending on whether the sample followed a normal distribution. For more than two groups, the ANOVA or the Kruskal-Wallis test was used to calculate significant differences. The correlation analysis was performed using Spearman’s test. The statistical difference is indicated by p-value (*P < 0.05, **P < 0.01, ***P < 0.001).

## Supplementary Material

Supplementray_Materials clean.doc

## Data Availability

All supporting data are available within the article and Supplemental Methods.
