# Supplementary material for: Gut microbiota alterations induced by Roux-en-Y gastric bypass result in glucose-lowering by enhancing intestinal glucose excretion
Source: Gut Microbes. 2025 Mar 3;17(1):2473519. doi: 10.1080/19490976.2025.2473519 (PMC11881838; doi:10.1080/19490976.2025.2473519)
Supplement: Supplementray_Materials clean.doc [file KGMI_A_2473519_SM5089.doc]

Supplementary Materials for

**Gut microbiota alterations induced by Roux-en-Y gastric bypass result in glucose-lowering by enhancing intestinal glucose excretion**

**
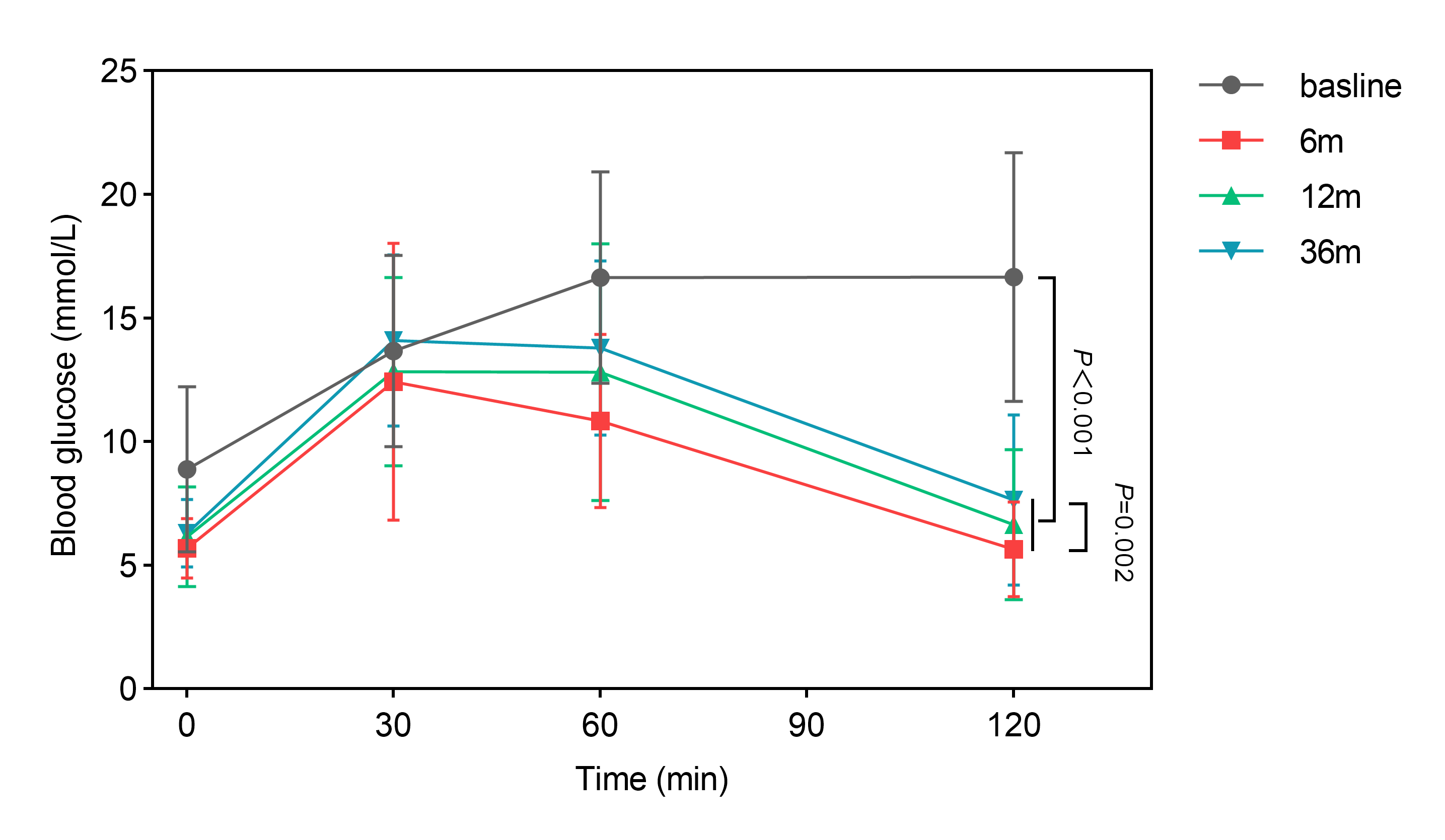
**

**Supplementary Figure 1. Improvement of glucose tolerance in T2DM patients after RYGB.**


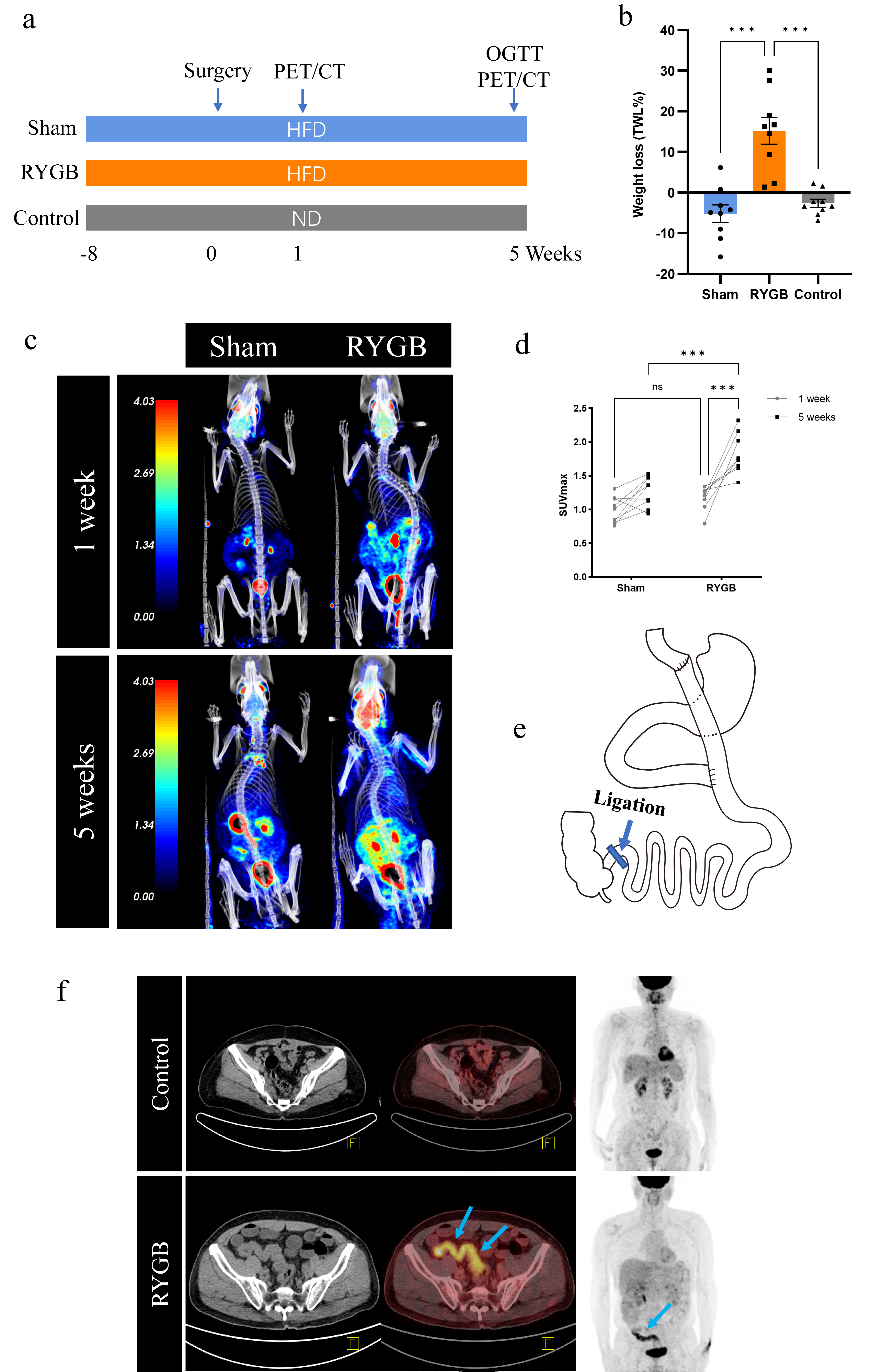


**Supplementary Figure 2.** **RYGB enhances intestinal glucose uptake in a time-dependent manner.** (a) Schematic diagram of animal experiment design. (b) Total weight loss after RYGB and sham in DIO rats (n=9 rat per group, one-way ANOVA). (c) Micro-PET/CT images 1 and 5 weeks after sham and RYGB, red and blue represent relatively higher and lower values, respectively. (d) SUV_max_ of intestinal FDG uptake at 1 and 5 weeks after sham and RYGB (n=9 rat per group, unpaired Student t-test). (e) The terminal ileum was ligated before the injection of FDG, to quantify the amount of glucose excreted into the small intestine and colon. (f) Representative images from whole-body 18F-FDG PET/CT scan of a newly diagnosed T2DM and a RYGB patient 13 years after surgery. The blue arrow indicates strong FDG uptake in the sigmoid of patients after RYGB. Data are presented as the mean ± SEM. *P＜0.05, **P＜0.01, ***P＜0.001.


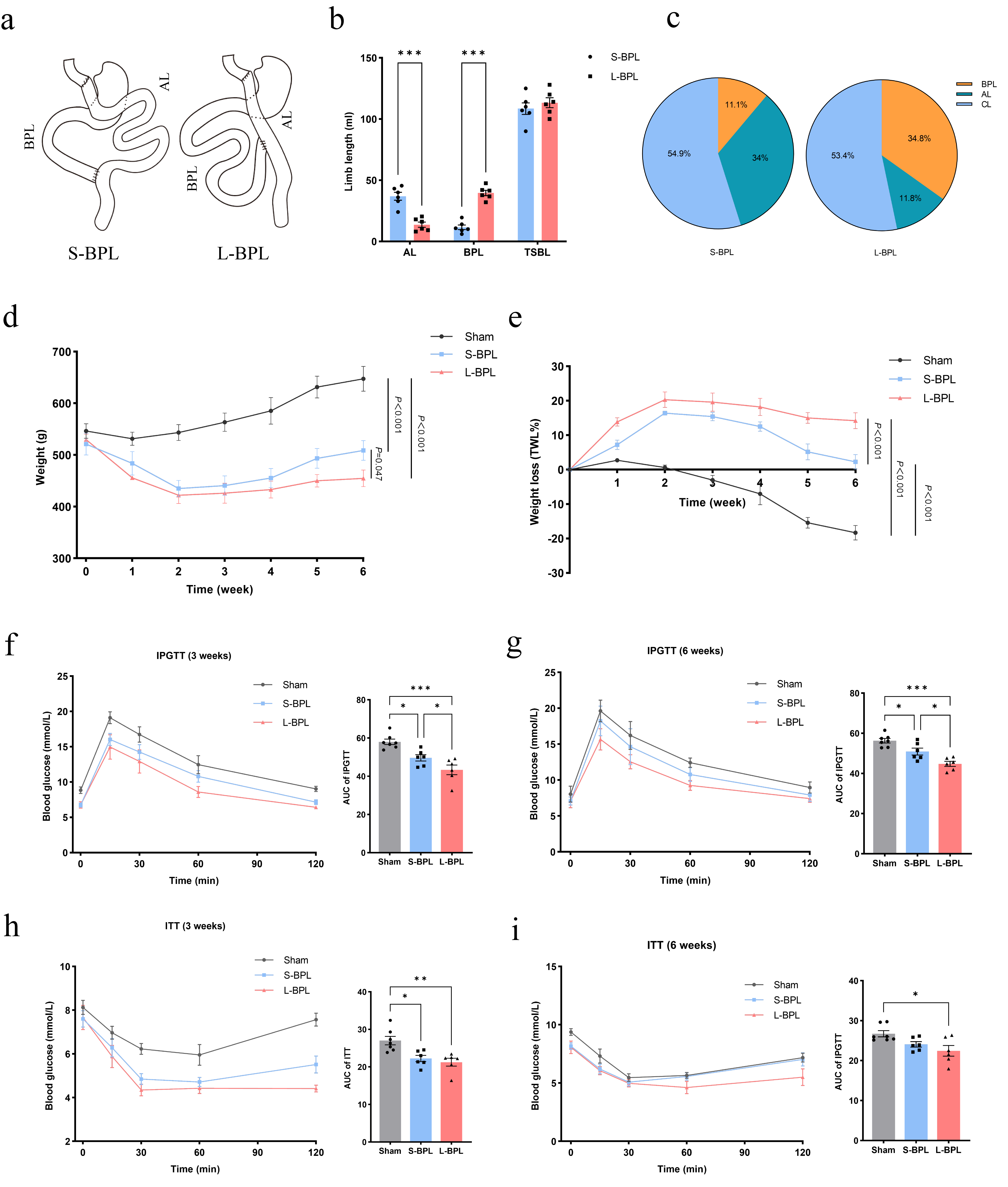


**Supplementary Figure 3. RYGB with a long BPL shows better glucose tolerance improvement after surgery.** (a) Surgery model for the S-BPL group and L-BPL group. (b) Limb lengths of AL, BPL, and TSBL (n=6 rat per group, two-way ANOVA). (c) Limb length proportions in the S-BPL group and L-BPL group. (d and e) Body weight (d) and TWL% (e) at 6 weeks after sham and RYGB with a long or short BPL (n=6-7 rat per group, two-way ANOVA). (f) Intraperitoneal glucose tolerance test (IPGTT) and area under the curve (AUC) at 3 weeks postoperatively (n=6-7 per group, one-way ANOVA). (g) IPGTT and AUC at 6 weeks postoperatively (n=6-7 per group, one-way ANOVA). (h) Insulin tolerance test (ITT) and area under the curve (AUC) at 3 weeks postoperatively (n=6-7 per group, one-way ANOVA). (i) ITT and AUC at 6 weeks postoperatively (n=6-7 per group, one-way ANOVA). Data are presented as the mean ± SEM. *P＜0.05, **P＜0.01, ***P＜0.001.


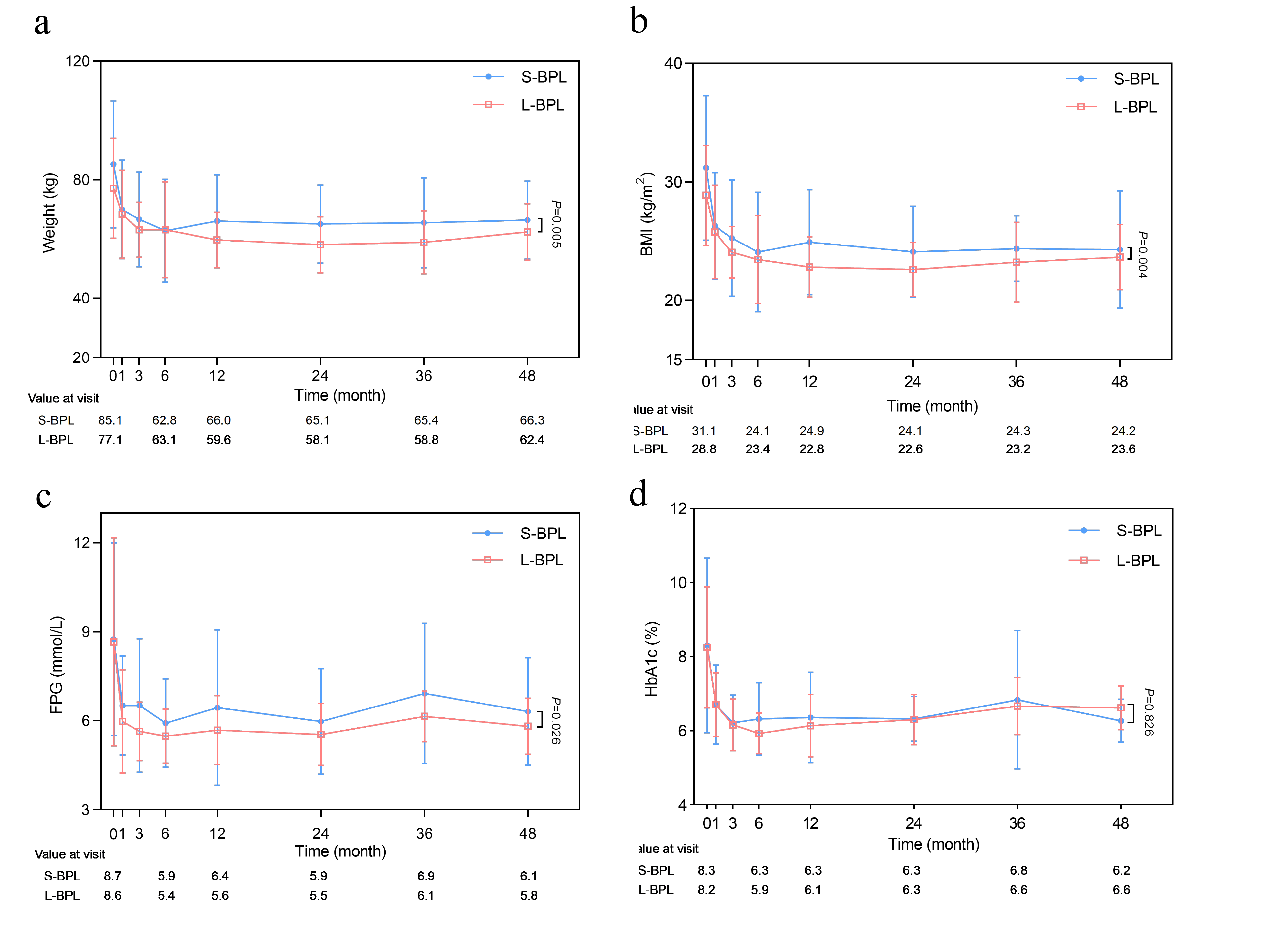
**Supplementary Figure 4. Changes in weight and glycemic control over 4 years after RYGB between S-BPL and L-BPL groups.** Body weight (a), BMI (b), FPG (c), and HbA1c (d) were compared between the two groups after surgery. Data are presented as the mean ± SD. P values for the overall comparisons were calculated with ANOVA.


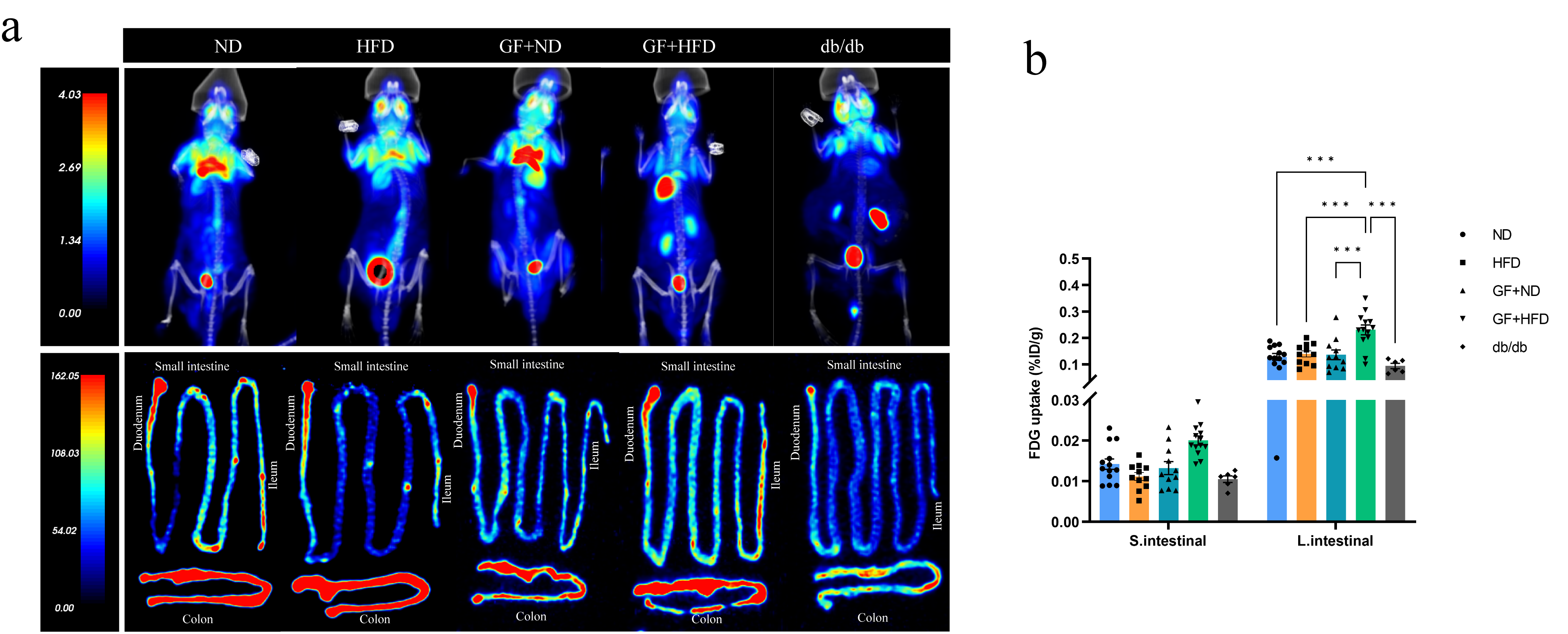
**Supplementary Figure 5.** Intestinal FDG uptake in multiple animal models. (a) Representative whole-body images and FDG uptake images of small intestine wall and colon wall of mice after PBS lavage in the ND, HFD, GF+ND, GF+HFD, and db/db groups, red and blue represent relatively higher and lower FDG accumulation, respectively. (b) FDG uptake (%ID/g) in small intestine wall and colon wall of mice in the ND, HFD, GF+ND, GF+HFD, and db/db groups after PBS lavage (n=6-13 mice per group, two-way ANOVA test). Data are shown as means ± SEM. *P＜0.05, **P＜0.01, ***P＜0.001.


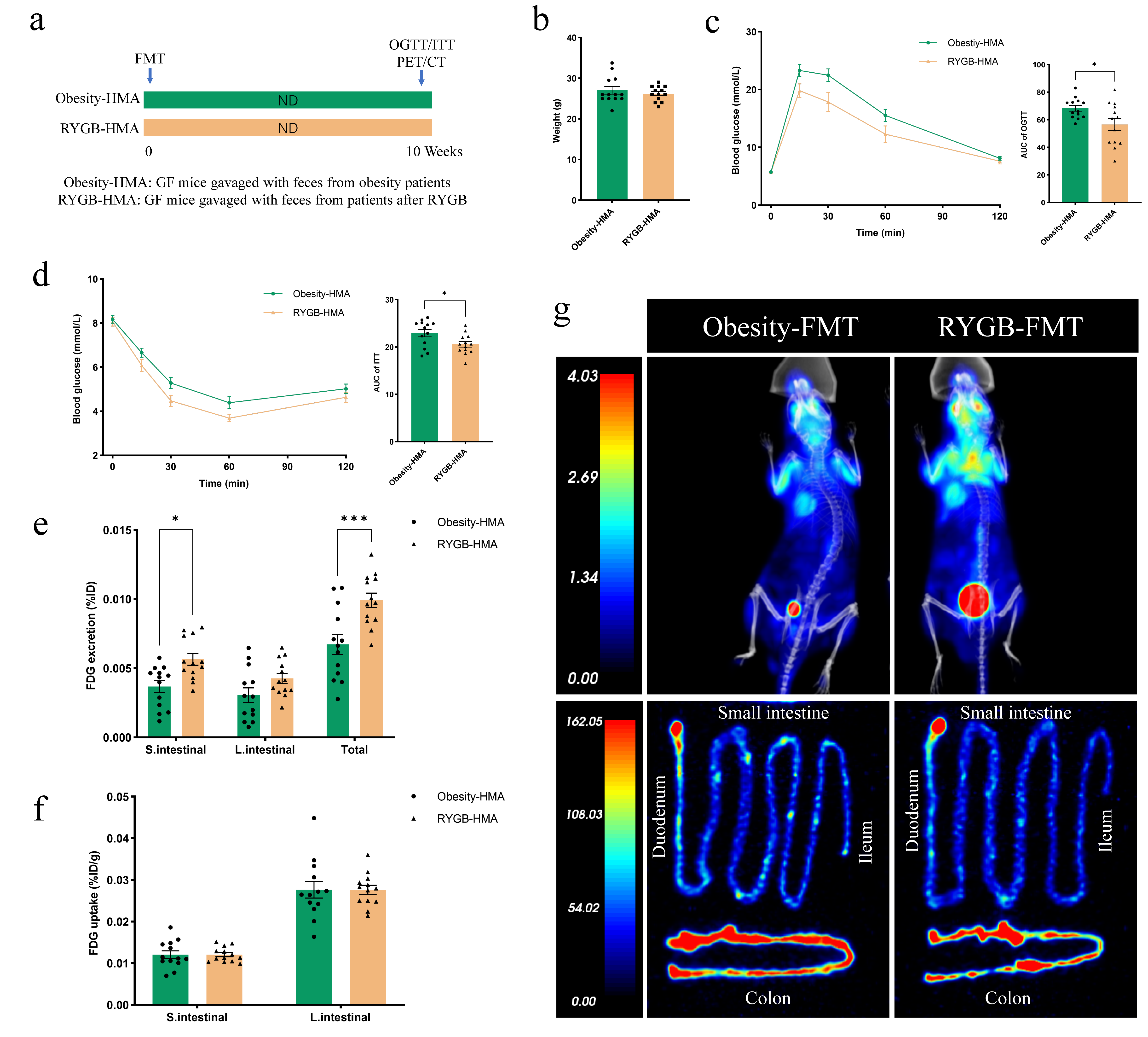
**Supplementary Figure 6.** **Enhanced intestinal FDG excretion in GF mice receiving fecal samples from patients after RYGB.** (a) Schematic diagram of animal experiment design. (b) Body weight of recipient mice in the Obesity and RYGB groups at 10 weeks after fecal microbiota transplantation (n=11-12 per group, unpaired Student t-test). (c) Oral glucose tolerance test (OGTT) and area under the curve (AUC) (n=13 per group. one-way ANOVA test). (d) Insulin tolerance test (ITT) and AUC (n=13 per group. one-way ANOVA test). (e) Intraluminal FDG excretion (%ID) in the small intestine and colon of mice in the Obesity-FMT and RYGB-FMT groups (n=13 per group. two-way ANOVA test). (f) FDG uptake in the small intestine wall and colon wall of mice in the Obesity-HMA and RYGB-HMA groups after PBS lavage (n=13 per group. two-way ANOVA test). (g) Representative whole-body and FDG uptake images of the small intestine wall and colon wall of mice after PBS lavage in the Obesity-HMA and RYGB-HMA groups.

**Supplementary Table 1. Preoperative characteristics of patients.** BMI, body mass index; OHT, oral hypoglycemic treatment; IT, insulin treatment; FPG, fasting plasma glucose; 2hPG, 2-hour postprandial blood glucose; HbA1c, glycated hemoglobin; HOMA-IR, homeostatic model assessment for insulin resistance.

| characteristic | Value (n=92) |
| --- | --- |
| Age (yr) | 43.76±10.44 |
| Male (Female) | 56(36) |
| Duration of diabetes (m) | 59.18±47.69 |
| Weight (kg) | 81.36±20.46 |
| BMI (kg/m^2^) | 30.02±5.50 |
| Medication use |  |
| OHT (%) | 36(39.1) |
| IT (%) | 15(16.3) |
| OHT+IT | 20(21.7) |
| FPG | 8.84±3.36 |
| 2hPG (mmol/L) | 16.67±5.03 |
| HbA1c (%) | 8.35±2.02 |
| C-peptide (ng/ml) | 1.43±1.44 |
| HOMA-IR | 5.00±6.96 |

**Supplementary Table 2. Comparison of preoperative factors in patients with or without achieved 20% TWL 1-year after surgery.** TWL, total weight loss; BMI, body mass index; OHT, oral hypoglycemic treatment; IT, insulin treatment; FPG, fasting plasma glucose; 2hPG, 2-hour postprandial blood glucose; HbA1c, glycated hemoglobin; HOMA-IR, homeostatic model assessment for insulin resistance.

| Characteristic | **＜20% TWL group**  (n=36) | **＞20% TWL group**  (n=32) | p value |
| --- | --- | --- | --- |
| Male（female） | （22/14） | （16/16） | 0.357 |
| Age（years） | 44.31±9.38 | 46.66±9.93 | 0.483 |
| Duration of diabetes (years) | 57.06±43.2 | 61.59±53.32 | 0.795 |
| Weight (kg) | 77.01±19.85 | 80.78±17.21 | 0.530 |
| BMI (kg/m2) | 28.09±4.84 | 31.48±5.64 | 0.055 |
| Medication use |  |  | 0.849 |
| OHT(%) | 15 | 13 |  |
| IT(%) | 7 | 4 |  |
| OHT+IT(%) | 9 | 7 |  |
| FPG (mmol/L) | 8.73±3.04 | 8.60±3.0 | 0.868 |
| 2hPG (mmol/L) | 17.43±5.08 | 16.09±5.03 | 0.483 |
| HbA1c (%) | 8.54±1.89 | 7.9±2.02 | 0.413 |
| C-peptide (ng/ml) | 1.12±0.92 | 1.75±1.71 | 0.178 |
| HOMA-IR | 3.07±3.33 | 6.12±6.17 | 0.055 |

**Supplementary Table 3. Comparison of baseline characteristics of patients in the S-BPL and L-BPL groups.** BPL, biliopancreatic limb; BMI, body mass index; OHT, oral hypoglycemic treatment; IT, insulin treatment; FPG, fasting plasma glucose; 2hPG, 2-hour postprandial blood glucose; HbA1c, glycated hemoglobin; HOMA-IR, homeostatic model assessment for insulin resistance. *P < 0.05.

| Characteristic | 1. BPL group   (n=45) | 1. BPL group   (n=47) | p value |
| --- | --- | --- | --- |
| Male（female） | （29/16） | （26/21） | 0.372 |
| Age（years） | 43.3±11.27 | 44.59±9.73 | 0.566 |
| Duration of diabetes (years) | 45.91±42.44 | 70.41±48.03 | 0.011* |
| Weight (kg) | 85.18 ± 21.43 | 77.09±16.87 | 0.051 |
| BMI (kg/m2) | 31.18 ± 6.11 | 28.85±4.21 | 0.039* |
| Medication use |  |  | 0.486 |
| OHT(%) | 17(42.2) | 19(40.4) |  |
| IT(%) | 5(11.1) | 10(21.2) |  |
| OHT+IT(%) | 9(20) | 12(25.5) |  |
| FPG (mmol/L) | 8.75±3.25 | 8.66±3.51 | 0.902 |
| 2hPG (mmol/L) | 15.7±5.15 | 17.47±4.78 | 0.104 |
| HbA1c (%) | 8.31±2.36 | 8.26±1.64 | 0.909 |
| C-peptide (ng/ml) | 1.79±1.92 | 1.12±0.65 | 0.035* |
| HOMA-IR | 5.26±8.38 | 4.52±5.15 | 0.629 |

**Supplementary Table 4. Donor patient characteristics.**

Pre-op refers to measurements before surgery, post-op refers to measurement at the follow-up time after RYGB.

|  | Obesity | | | | | RYGB | | | | |  |
| --- | --- | --- | --- | --- | --- | --- | --- | --- | --- | --- | --- |
| Biometrics | 1 | 2 | 3 | mean | SD | 1 | 2 | 4 | mean | SD | p values |
| Age (year) | 31 | 24 | 36 | 30.33 | 6.03 | 25 | 42 | 27 | 31.33 | 9.29 | 1.52 |
| Male (female) | female | female | female |  |  | Male | female | Female |  |  |  |
| Post-op follow-up time (year) |  |  |  |  |  | 5 | 7 | 9 |  |  |  |
| Pre-op Weight (kg) | 132.1 | 97.1 | 101.6 | 110.27 | 19.04 | 145 | 91.6 | 83 | 106.53 | 33.59 | 0.88 |
| Post-op Weight (kg) |  |  |  |  |  | 115 | 71.5 | 68 | 84.83 | 26.18 |  |
| Pre-op BMI (kg/m^2^) | 51.1 | 37.9 | 39.9 | 42.97 | 7.11 | 44.26 | 38.1 | 31.5 | 37.95 | 6.38 | 0.41 |
| Post-op BMI (kg/m^2^) |  |  |  |  |  | 34.33 | 29.38 | 25.9 | 29.87 | 4.24 |  |
| Pre-op FPG (mmol/L) | 7.54 | 5.78 | 8.93 | 7.42 | 1.58 | 7.06 | 10.61 | 5.89 | 7.85 | 2.46 | 0.81 |
| Post-op FPG (mmol/L) |  |  |  |  |  | 5.1 | 4.78 | 5.14 | 5.01 | 0.20 |  |
| Pre-op HbA1C (%) | 8.3 | 6.3 | 7.8 | 7.47 | 1.04 | 7.1 | 14 | 5.7 | 8.93 | 4.44 | 0.61 |
| Post-op HbA1C (%) |  |  |  |  |  | 5.5 | 6.4 | 5.5 | 5.80 | 0.52 |  |
| Pre-op C-peptide (ng/ml) | 4.48 | 6.09 | 3.95 | 4.84 | 1.11 | 2.66 | 1.47 | 4.25 | 2.79 | 1.39 | 0.12 |
| Post-op C-peptide (ng/ml) |  |  |  |  |  | 2.08 | 4.99 | 1.07 | 2.71 | 2.04 |  |
| Pre-op INS (pmol/L) | 170 | 429.2 | 163.1 | 254.10 | 151.68 | 61.14 | 13.68 | 30.16 | 34.99 | 24.10 | 0.07 |
| Post-op INS (pmol/L) |  |  |  |  |  | 133.2 | 133.4 | 5.39 | 90.66 | 73.85 |  |
| Pre-op TC (mmol/L) | 4.01 | 5.58 | 5.86 | 5.15 | 1.00 | 4.31 | 4.11 | 3.66 | 4.03 | 0.33 | 0.14 |
| Post-op TC (mmol/L) |  |  |  |  |  | 3.37 | 4.99 | 4.19 | 4.18 | 0.81 |  |
| Pre-op TG (mmol/L) | 1.29 | 1.69 | 2.15 | 1.71 | 0.43 | 2.81 | 2.84 | 1.65 | 2.43 | 0.68 | 0.19 |
| Post-op TG (mmol/L) |  |  |  |  |  | 1.69 | 2.17 | 0.99 | 1.62 | 0.59 |  |
| Pre-op LDL-c (mmol/L) | 2.27 | 3.54 | 3.78 | 3.20 | 0.81 | 2.91 | 2.83 | 2.39 | 2.71 | 0.28 | 0.38 |
| Post-op LDL-c (mmol/L) |  |  |  |  |  | 2.07 | 3.31 | 2.86 | 2.75 | 0.63 |  |
| Pre-op HDL-c (mmol/L) | 1.36 | 1.01 | 1.13 | 1.17 | 0.18 | 0.86 | 0.73 | 0.48 | 0.69 | 0.19 | 0.03 |
| Post-op HDL-c (mmol/L) |  |  |  |  |  | 0.96 | 1.19 | 1.02 | 1.05 | 0.08 |  |
